# Immunometabolism in animal production: building efficiency from health

**DOI:** 10.1093/af/vfac065

**Published:** 2022-10-14

**Authors:** Rafael Alejandro Palladino, Dario Colombatto

**Affiliations:** Faculty of Agricultural Science—UNLZ, CONICET, Lomas de Zamora, Buenos Aires, Argentina; Facultad de Agronomía, CONICET/University of Buenos Aires INPA, Buenos Aires, Argentina

In the last decades, individual animal production increased dramatically as consequence of several improvements related to management, genetics, nutrition and health, among others. From a biological point of view, the increase in efficiency and production is a huge challenge that, under some circumstances, can negatively affect animal health and consequently animal well-being and production. Immunometabolism, defined as an interplay between immunological and metabolic processes (Mathis and Shoelsen, 2011), is an emerging field of research that may help animal scientists to better understand the link between production and health. In other words, immunometabolism studies how nutrients modulate the immune response.

Improving health may affect positively the economic results in all livestock industries. Therefore, understanding the mechanism underlying the metabolism of the immune system is critical for shaping feeding and management strategies. This Special Issue aims to summarize new knowledge and perspectives on this area, consolidating some concepts previously developed, while bringing new questions for future research.

From a perspective view, Davis (2022) introduces the concept of the immune system not only as a host defense mechanism but also as a tool for keeping homeostasis. This shows an unconventional definition that, however, the author remarks as conceptually right since the immune activity is, at the end of the day, a reaction to a perturbation in the host’s physiological steady state. The author also highlights that the immune system activation brings an energy cost to the animal, re-addressing the nutrients from growth and reproduction purposes to those mechanisms that help the organism to survive. Inflammation is recognized as the process that a certain point manages the resources of the nutrients for growth and survival. Avoiding inflammation may enhance productivity but, for instance, a certain level of inflammatory activation is necessary for immune system development or onset of lactation. Finally, whether immunological activation during early life affects future production is a question that remains uncertain.

Another interesting point is added by Elsasser (2022), who presents the fact that microbes highly interact with the animal host. Using the chicken (*Gallus gallus*) as an animal model, the author shows how the gut microbial population “talks” to the host endocrine/immune system, to regulate the nutrient availability for different tissues under good conditions of health or stress. In this sense, there is evidence that specific changes in bacterial population and bioactive compounds modify and modulate the activation of certain types of host cells to secrete factors that disfavor microbes related to dysbiosis, inflammation, pathogenesis, and disease (Elsasser, 2022).

Alfaro and Moisa (2022) address the problem of fescue toxicosis, caused by the consumption of tall fescue with an endophyte called *Epichloë coenophiala*. This endophyte is well known worldwide due to its massive negative economic impact in cattle. The consumption of the ergot alkaloids produced by the endophyte is toxic for grazing animals, reducing feed intake, body weight, etc. Some of these ergot alkaloids are known to be vasoconstrictors, leading to a reduction of blood flow to reproductive organs (reducing reproductive performance). In addition, it reduces prolactin synthesis, which indeed reduces milk production and offspring body weight. The way these secondary metabolites interact with the animal at different levels, including rumen and gut microbiome, and liver metabolism, is approached by the authors, showing an interesting example of how external stressors activate the immune system and negatively affect production.

Dairy cows are challenged from the biological point of view since genetic and management improvements resulted in high levels of production. Leoni et al. (2022) focus their review on how feeding cows properly may enhance transition from gestation to lactation through a control of inflammation, leading to a reduction of potential health issues and increasing milk production during the entire lactation. The authors clearly state the connection between gut barrier integrity and activation of the immune system. Feeding strategies aimed to improve gut health may reduce inflammation and consequently, the metabolic cost that impairs production. Therefore, some directions and suggestions are shown in order to enhance health and productivity in transition dairy cows.

In the same line, Zachut et al. (2022) introduce the role of lipid mediators as immunomodulators, affecting inflammation during transition in dairy cows and the possibility of using these molecules to manipulate the metabolism in order to improve animal health and productivity. Also, the authors state the importance of adipose tissue not as an energy store but as an important endocrine tissue that regulates immune activation and inflammation response. The role of the endocannabinoid system, the fatty acids released during mobilization, and oxi-lipids produced at cellular level is well described, showing a link between the energetic metabolism and the immune system activation. The understanding of the lipid metabolism during transition lead to the development of nutritional strategies capable of reducing inflammation through supplementation with immunomodulators such as omega-3 fatty acids or conjugated linoleic acid. Another strategy proposed by Zachut et al. (2022) is the supplementation of rumen-protected niacin, which is involved in the inhibition of lipolysis, reducing the risk of immunometabolism dysfunction, also supporting the idea that reducing adipose tissue mobilization may reduce inflammation.

Loor and Elolimy (2022) focus their review on the role of transcription factors and nutrients that trigger the immunometabolism activation. Although most of the information is available for transition dairy cows, mechanisms involved in these processes can be extrapolated to other physiological states and species. The authors also studied the role of the gut microbiome and its link with the immunometabolism, especially during the early stages of growth, where microbial colonization contributes to shape the gut morphology and immune system development. A deeper understanding of the factors affecting and triggering the immune activation may bring new tools for modulating the animal physiology, thereby enhancing productivity. In this line, increasing the knowledge of the gut microbiota physiology and its link with the intestinal epithelium metabolism will lead to novel feeding strategies that may enhance animal health and productivity.

As previously mentioned, the main goal of this issue of *Animal Frontiers* is to summarize the current knowledge on the field of immunometabolism, together with the identification of the next probable challenges and opportunities that may exist on this topic. A better understanding of these mechanisms will help the livestock industry to increase productivity and efficiency, while improving animal welfare.

## About the Authors

**Rafael Alejandro Palladino** is a researcher at CONICET (National Scientific and Technical Research Council) and also a professor at University of Lomas de Zamora, Argentina, where he teaches animal nutrition. He is a co-founder of Smart Farming Consulting. His research is focused on the interaction between nutrition and the immune system, mainly using transition dairy cows as animal models. Currently, Palladino is supervising several PhD students. Palladino is a former editor of the Argentinean Journal of Animal Production (Revista Argentina de Producción Animal) and serves as a reviewer in several peer review journals in the field of animal sciences.

**Darío Colombatto** is a professor at The University of Buenos Aires, Argentina, where he teaches and does research on Beef Cattle Production Systems. He also belongs to the CONICET (National Scientific and Technical Research Council), where he is currently supervising several MSc and PhD students, associated with several Universities in Argentina. He served as a President of the Argentinean Society of Animal Production from 2007 to 2009 and was a Senior Editorial Board Member of the Animal Feed Science and Technology. His research interests range from studies on feed additives as ruminal and intestinal modifiers, mitigation of greenhouse gas emissions from cattle, and simulation models of whole production systems.
